# Crystal structure of the tetra­hydro­furan disolvate of a 94:6 solid solution of [*N* ^2^,*N* ^6^-bis­(di-*tert*-butyl­phosphan­yl)pyridine-2,6-di­amine]­dibromido­manganese(II) and its monophosphine oxide analogue

**DOI:** 10.1107/S2056989017011276

**Published:** 2017-08-08

**Authors:** Markus Rotter, Matthias Mastalir, Mathias Glatz, Berthold Stöger, Karl Kirchner

**Affiliations:** a Institute of Applied Synthetic Chemistry, TU Wien, Getreidemarkt 9/163, A-1060 Vienna, Austria; b X-Ray Centre, TU Wien, Getreidemarkt 9, A-1060 Vienna, Austria

**Keywords:** crystal structure, PNP, manganese, pincer complex, solid solution, phosphine oxide

## Abstract

The MnBr_2_ complex of *N* ^2^,*N* ^6^-bis­(di-*tert*-butyl­phosphan­yl)pyridine-2,6-di­amine (**1**·MnBr_2_) co-crystallizes with 5.69% of the monophosphine oxide analogue (**1**O·MnBr_2_) and two tetra­hydro­furan (THF) mol­ecules, namely [*N* ^2^,*N* ^6^-bis­(di-*tert*-butyl­phosphan­yl)pyridine-2,6-di­amine]­dibromido­manganese(II)–[bis­(di-*tert*-butyl­phosphan­yl)({6-[(di-*tert*-butyl­phosphan­yl)amino]­pyridin-2-yl}amino)­phosphine oxide]di­bromido­manganese(II)–tetra­hydro­furan (0.94/0.06/2), [MnBr_2_(C_21_H_41_N_3_P_2_)]_0.94_[MnBr_2_(C_21_H_41_N_3_OP_2_)]_0.06_·2C_4_H_8_O. The **1**·MnBr_2_ and **1**O·MnBr_2_ complexes are occupationally disordered about general positions. Both complexes feature square-pyramidal coordination of the Mn^II^ atoms. They are connected by weak N—H⋯Br hydrogen bonding into chains extending along [001]. The THF mol­ecules are located between the layers formed by these chains. One THF mol­ecule is involved in hydrogen bonding to an amine H atom.

## Abstract 2

The MnBr_2_ complex of *N* ^2^,*N* ^6^-bis­(di-*tert*-butyl­phosphan­yl)pyridine-2,6-di­amine (**1**·MnBr_2_) co-crystallizes with 5.69% of the monophosphine oxide analogue (**1**O·MnBr_2_) and two tetra­hydro­furan (THF) mol­ecules, namely {*N* ^2^,*N* ^6^-bis­(di-*tert*-butyl­phosphan­yl)pyridine-2,6-di­amine}­dibromido­manganese(II)–[bis­(di-*tert*-butyl­phosphan­yl)({6-[(di-*tert*-butyl­phosphan­yl)amino]­pyridin-2-yl}amino)­phosphine oxide]di­bromido­manganese(II)–tetra­hydro­furan (0.94/0.06/2), [MnBr_2_(C_21_H_41_N_3_P_2_)]_0.94_[MnBr_2_(C_21_H_41_N_3_OP_2_)]_0.06_·2C_4_H_8_O. The **1**·MnBr_2_ and **1**O·MnBr_2_ complexes are connected by weak N—H⋯Br hydrogen bonding into chains extending along [001] with the THF mol­ecules located between the layers formed by these chains.

## Chemical context   

Pincer complexes of transition metals are versatile homogeneous catalysts (Dobereiner & Crabtree, 2010 ▸; Mastalir *et al.*, 2017*a* ▸). Traditionally, platinum-group metal complexes have been employed in these applications (Zell & Milstein, 2015 ▸; Bähn *et al.*, 2011 ▸; Crabtree *et al.*, 2011 ▸; Watson & Williams, 2010 ▸; Gunanathan *et al.*, 2007 ▸; Zhang *et al.*, 2005 ▸; Michlik & Kempe, 2010 ▸; Michlik *et al.*, 2012 ▸). Our group is dedicated to the development of more cost-effective and environmentally friendly alternatives, such as PNP (pincer ligand coordinating *via* P, N and P) complexes of Fe (Glatz *et al.*, 2015*a* ▸,*b* ▸; Mastalir *et al.*, 2016*a* ▸). Recently, we extended our research scope to Mn^I^ PNP complexes (Mastalir *et al.*, 2016*b* ▸,*c* ▸, 2017*b* ▸).

In this context, we attempted the synthesis of the MnBr_2_ complex with the PNP ligand *N* ^2^,*N* ^6^-bis­(di-*tert*-butyl­phosphan­yl)pyridine-2,6-di­amine (**1**) as a precursor to Mn^I^ complexes. Inadvertently, on recrystallization of the crude product, a 94.31:5.69 (14)% solid solution of the expected **1**·MnBr_2_ and its phosphine oxide analogue **1**O·MnBr_2_ co-crystallized with two THF solvent mol­ecules (see scheme), most likely as a result of an impure starting ligand. The crystal under investigation accordingly has the composition 0.9431(**1**·MnBr_2_)·0.0569(**1**O·MnBr_2_)·2THF.
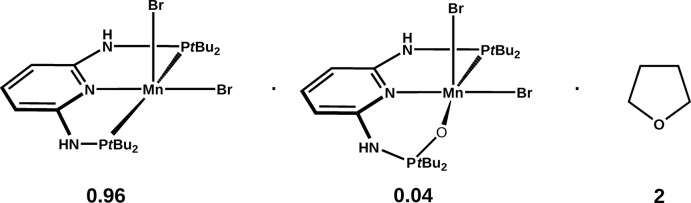


## Structural commentary   

The title crystal possesses *P*2_1_/*c* symmetry. A 94.31:5.69 (14) overlay of the **1**·MnBr_2_ complex and the corresponding mono-oxidized **1**O·MnBr_2_ complex is located on general positions. Two crystallographically independent THF solvent mol­ecules are likewise located on general positions, one of which is positionally disordered.

The ligands of both the non-oxidized and the oxidized complexes occupy virtually the same space. They could therefore not be resolved into distinct sites and even the atomic displacement parameters (ADPs) are not significantly enlarged. The Mn and Br atoms, on the other hand, are clearly separated within the resolution of the experiment.

**Figure 1:**
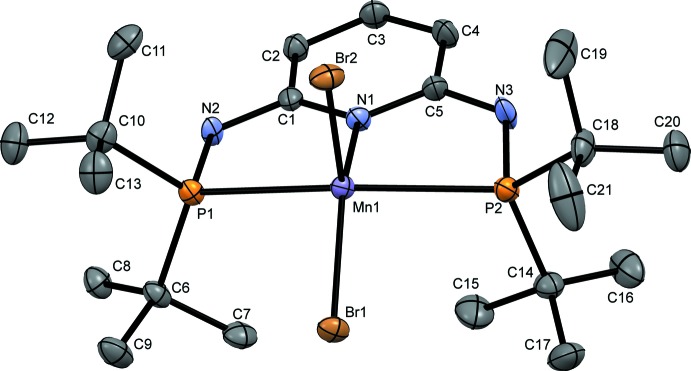
The mol­ecular structure of **1**·MnBr_2_. C (grey), N (blue), P and Br (orange), and Mn (purple) atoms are represented by ellipsoids drawn at the 50% probability levels. H atoms have been omitted for clarity.

The Mn^II^ atom of the non-oxidized **1**·MnBr_2_ complex features fivefold coordination with the PNP-ligand and two bromine atoms (Fig. 1 ▸) in a square-pyramidal conformation with a τ5 parameter (Addison *et al.*, 1984 ▸) of 0.083. The ideal τ5 values for square-pyramidal and trigonal–bipyramidal coordinations are 0 and 1, respectively. The Mn atom is nearly equidistant [2.644 (9) and 2.639 (10) Å] to both P atoms.

The complex adopts a distinctly non-planar configuration with distances to the least-squares (LS) plane defined by the pryidine ring and amine-N atoms of 0.4391 (7) Å (Mn), 0.0700 (7) Å (P1) and 0.3100 (7) Å (P2), as is characteristic for this class of compounds.

In comparison, the recently structurally characterized MnCl_2_ complex of the isopropyl analogue of **1** (Mastalir *et al.*, 2017*a* ▸) features an even more ideal square-pyramidal conformation (τ5 = 0.041) and the Mn^II^ atom is likewise nearly equidistant to both P atoms [2.593 (5) and 2.579 (5) Å]. Likewise, the deviation from planarity is in the same range [distances to the LS plane described above: 0.4158 (2) Å (Mn), 0.3190 (4) Å (P1) and 0.0334 (4) Å (P2)].

**Figure 2:**
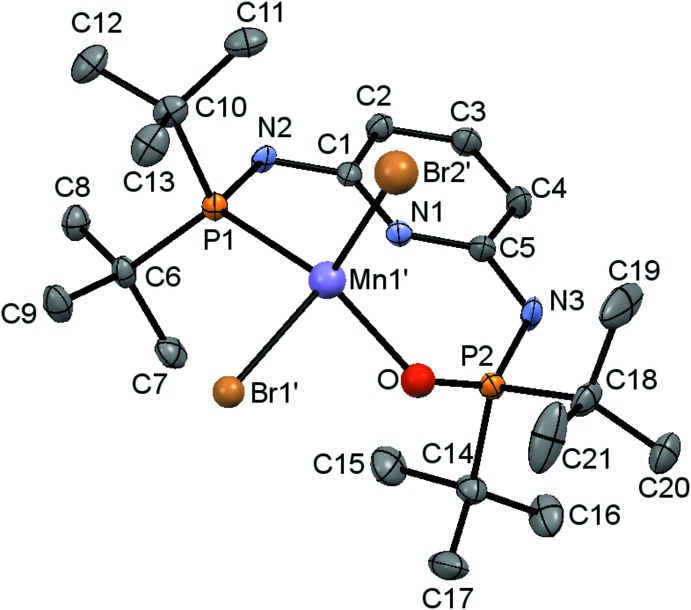
The mol­ecular structure of **1**O·MnBr_2_. Atom colour codes as in Fig. 1 ▸ with O (red).

**Figure 3:**
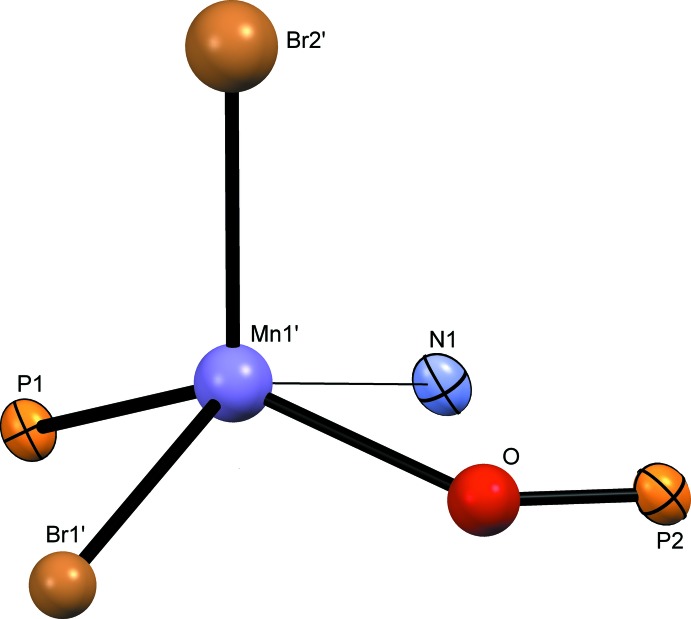
The coordination of the Mn atom in **1**O·MnBr_2_. Atom colour codes as in Figs. 1 ▸ and 2 ▸.

In the monooxidized **1**O·MnBr_2_ complex (Fig. 2 ▸), the coordination deviates more from the square-pyramidal mode than in **1**·MnBr_2_ (τ5 = 0.196; Fig. 3 ▸). The O atom introduces an additional distortion, leading to an increased deviation from planarity, whereby the Mn′ and O atoms are located on opposite sides of the LS plane described above [0.712 (13) Å (Mn) and 0.12 (4) Å (O)]. The Mn′—P1 bond is distinctly shorter [2.453 (12) Å] than the corresponding bond in the non-oxidized complex.

## Supra­molecular features   

**Figure 4:**
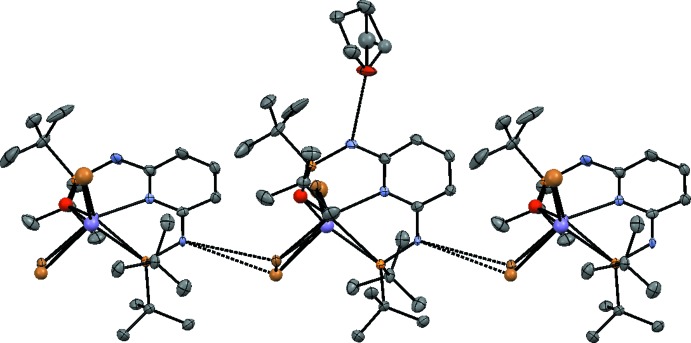
Inter­molecular hydrogen bonding (dashed lines) in the title crystal. Complexes are shown as an overlay of **1**·MnBr_2_ and **1**O·MnBr_2_. Atom colour codes as in Figs. 1 ▸ and 2 ▸.

The disordered THF mol­ecule (O1/C22–C25) is connected to a complex mol­ecule *via* a strong N1—H⋯O1 hydrogen bond (Table 1 ▸). The second THF mol­ecule is not involved in hydrogen bonding (Fig. 4 ▸).

The amine functionality that is not bonded to THF connects *via* a weak N2—H⋯Br1(Br1′) hydrogen bond, thus forming infinite chains of complex mol­ecules extending along [001]. Adjacent complexes in this chain are related by the *c* glide reflection.

**Figure 5:**
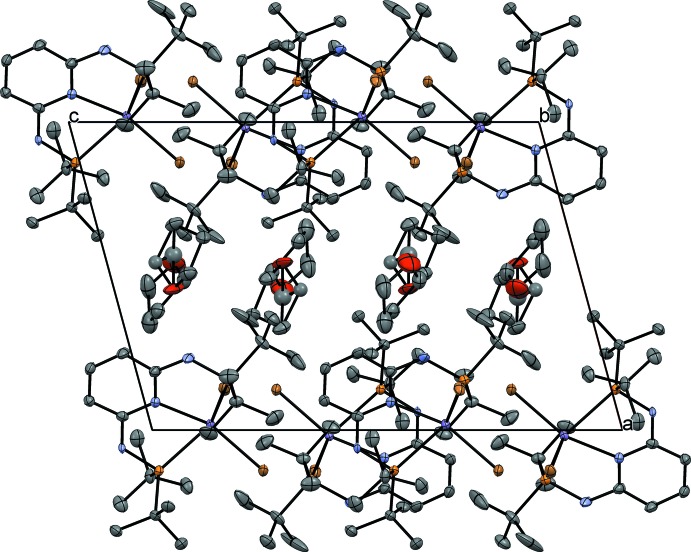
Packing plot of the title crystal looking along [010].

No further bonding inter­molecular inter­actions are observed in the crystal structure. The chains of complexes contact in the [001] direction *via* van der Waals inter­actions, forming distinct layers parallel to (100). Between these layers are located the hydrogen-bonded and free THF mol­ecules (Fig. 5 ▸).

## Database survey   

A search in the Cambridge Structural Database (Version 5.37; last update March 2016; Groom *et al.*, 2016 ▸) for structures of fivefold-coordinated Mn/PNP complexes revealed no entries. Nevertheless, our group recently published the MnCl_2_ complex of the isopropyl analogue of **1** (see above). Moreover, three related Mn(PNP)(CO)_3_ complexes with octa­hedral coordination modes are known. One of these compounds is likewise pyridine-based (Flörke & Haupt, 1991 ▸), whereas the others are based on ditolyl­amines (Radosevich *et al.*, 2009 ▸). No ligand mono-oxidized analogues of Mn/PNP complexes have been described up to now.

## Synthesis and crystallization   

The synthesis of **1** was performed as described previously (Deibl & Kempe, 2016 ▸). THF was dried over Na under an Ar atmosphere. All other reagents were obtained commercially and used as received. **1** and MnBr_2_ were stirred in dry THF for 18 h under an Ar atmosphere (see reaction scheme). The complex **1**·MnBr_2_ was precipitated by addition of *n*-pentane. The microcrystalline powder was washed twice with *n*-pentane. Crystals were grown by slow vapour diffusion of diethyl ether into a room-temperature saturated solution of **1**·MnBr_2_ in THF.
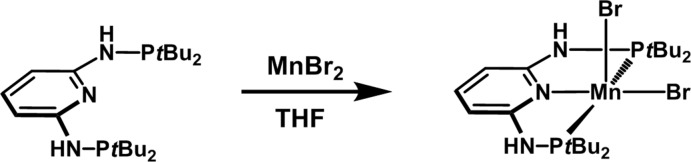


## Refinement   

Crystal data, data collection and structure refinement details are summarized in Table 2 ▸. H atoms bonded to C atoms were placed in calculated positions and refined as riding atoms, with fixed bond lengths in the range 0.95–1.00 Å and *U*_iso_(H) = 1.2*U*_eq_(C) or 1.5*U*_eq_(C_Me_). The two amine H atoms were located in difference-Fourier maps and were refined freely.

Excessive electron density in difference-Fourier maps was attributed to alternative positions of the Mn and Br atoms. The Mn and Br atoms were therefore refined as positionally disordered (minor positions: Mn′ and Br′). The occupancies of the atoms of both orientations were constrained to the same value and the sum of the occupancies of both orientations were constrained to 1. The atoms in the minor (*ca* 6%) orientation were modelled with isotropic ADPs. The minor orientation featured an unreasonably long Mn—P distance (*ca* 3.18 Å). Inspection of the electron density in the difference-Fourier map close to the P atom revealed a faint positive peak that was attributed to an O atom that is bound to the P atom, forming an phosphine oxide. The occupancy of this atom was constrained to be equal to the occupancy of the minor positions. The position of the additional O atom was refined freely.

A C atom of a THF mol­ecule featured excessively anisotropic ADPs. The position was therefore split and refined as positionally disordered with the sum of the occupancies of both positions constrained to 1; occupancy ratio 0.526 (14):0.474 (14). Both C atoms were refined with isotropic ADPs.

## Supplementary Material

Crystal structure: contains datablock(s) I, global. DOI: 10.1107/S2056989017011276/sj5533sup1.cif

Structure factors: contains datablock(s) I. DOI: 10.1107/S2056989017011276/sj5533Isup2.hkl

CCDC reference: 1565992

Additional supporting information:  crystallographic information; 3D view; checkCIF report

## Figures and Tables

**Table 1:** Hydrogen-bond geometry (Å, °)

*D*—H⋯*A*	*D*—H	H⋯*A*	*D*⋯*A*	*D*—H⋯*A*
N2—H1N2⋯Br1^i^	0.83 (3)	2.80 (3)	3.625 (2)	173 (2)
N2—H1N2⋯Br1′^i^	0.83 (3)	2.81 (3)	3.629 (7)	169 (3)
N3—H1N3⋯O1	0.78 (4)	2.22 (4)	2.990 (4)	171 (3)

Symmetry code: (i) 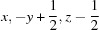.

**Table 2:** Experimental details

Crystal data	
Chemical formula	[MnBr_2_(C_21_H_41_N_3_P_2_)]_0.94_[MnBr_2_(C_21_H_41_N_3_OP_2_)]_0.06_·2C_4_H_8_O
*M*_r_	757.4
Crystal system, space group	Monoclinic, *P*2_1_/*c*
Temperature (K)	100
*a*, *b*, *c* (Å)	11.6496 (7), 18.5016 (11), 17.1626 (9)
β (°)	105.1763 (16)
*V* (Å^3^)	3570.2 (4)
*Z*	4
Radiation type	Mo *K*α
μ (mm^−1^)	2.73
Crystal size (mm)	0.45 × 0.43 × 0.42

Data collection	
Diffractometer	Bruker Kappa APEXII CCD
Absorption correction	Multi-scan (*SADABS*; Bruker, 2015 ▸)
*T*_min_, *T*_max_	0.29, 0.32
No. of measured, independent and observed [*I* > 3σ(*I*)] reflections	29339, 8452, 6175
*R*_int_	0.031
(sin θ/λ)_max_ (Å^−1^)	0.659

Refinement	
*R*[*F* > 3σ(*F*)], *wR*(*F*), *S*	0.039, 0.045, 1.92
No. of reflections	8452
No. of parameters	377
H-atom treatment	H atoms treated by a mixture of independent and constrained refinement
Δρ_max_, Δρ_min_ (e Å^−3^)	1.02, −0.70

Computer programs: *APEX2* and *SAINT-Plus* (Bruker, 2015 ▸), *SHELXT* (Sheldrick, 2015 ▸), *JANA20006* (Petříček *et al.*, 2014 ▸), *Mercury* (Macrae *et al.*, 2006 ▸) and *publCIF* (Westrip, 2010 ▸).

## supplementary crystallographic information

### Crystal data

**Table:**

[MnBr_2_(C_21_H_41_N_3_P_2_)]_0.94_[MnBr_2_(C_21_H_41_N_3_OP_2_)]_0.06_·2C_4_H_8_O	*F*(000) = 1574
*M**_r_* = 757.4	*D*_x_ = 1.409 Mg m^−^^3^
Monoclinic, *P*2_1_/*c*	Mo *K*α radiation, λ = 0.71073 Å
Hall symbol: -P 2ycb	Cell parameters from 9972 reflections
*a* = 11.6496 (7) Å	θ = 2.5–27.9°
*b* = 18.5016 (11) Å	µ = 2.73 mm^−^^1^
*c* = 17.1626 (9) Å	*T* = 100 K
β = 105.1763 (16)°	Block, colourless
*V* = 3570.2 (4) Å^3^	0.45 × 0.43 × 0.42 mm
*Z* = 4	

### Data collection

**Table:**

Bruker Kappa APEXII CCD diffractometer	8452 independent reflections
Radiation source: X-ray tube	6175 reflections with *I* > 3σ(*I*)
Graphite monochromator	*R*_int_ = 0.031
ω– and φ–scans	θ_max_ = 28.0°, θ_min_ = 1.7°
Absorption correction: multi-scan SADABS	*h* = −15→12
*T*_min_ = 0.29, *T*_max_ = 0.32	*k* = −24→24
29339 measured reflections	*l* = −22→21

### Refinement

**Table:**

Refinement on *F*	250 constraints
*R*[*F* > 3σ(*F*)] = 0.039	H atoms treated by a mixture of independent and constrained refinement
*wR*(*F*) = 0.045	Weighting scheme based on measured s.u.'s *w* = 1/(σ^2^(*F*) + 0.0001*F*^2^)
*S* = 1.92	(Δ/σ)_max_ = 0.033
8452 reflections	Δρ_max_ = 1.02 e Å^−^^3^
377 parameters	Δρ_min_ = −0.70 e Å^−^^3^
0 restraints	

### Fractional atomic coordinates and isotropic or equivalent isotropic displacement parameters (Å^2^)

**Table:**

	*x*	*y*	*z*	*U*_iso_*/*U*_eq_	Occ. (<1)
Br1	−0.13106 (3)	0.21675 (2)	0.212868 (18)	0.02007 (12)	0.9431 (14)
Br2	0.13368 (4)	0.33931 (4)	0.17703 (3)	0.02331 (15)	0.9431 (14)
Mn1	0.01885 (5)	0.22707 (4)	0.12718 (3)	0.01482 (17)	0.9431 (14)
P1	−0.13202 (6)	0.26779 (4)	−0.00936 (4)	0.0159 (2)	
P2	0.16177 (6)	0.11993 (4)	0.19188 (4)	0.0181 (2)	
N1	0.0909 (2)	0.17720 (13)	0.02291 (13)	0.0176 (8)	
N2	−0.0580 (2)	0.23660 (13)	−0.07467 (14)	0.0162 (8)	
N3	0.2335 (2)	0.11176 (17)	0.11855 (16)	0.0304 (10)	
C1	0.0446 (2)	0.19576 (15)	−0.05555 (17)	0.0171 (9)	
C2	0.0954 (2)	0.17562 (16)	−0.11712 (17)	0.0196 (10)	
C3	0.1999 (3)	0.13664 (16)	−0.09629 (18)	0.0230 (10)	
C4	0.2492 (3)	0.11602 (18)	−0.01763 (18)	0.0270 (11)	
C5	0.1901 (3)	0.13566 (17)	0.04007 (17)	0.0221 (10)	
C6	−0.2769 (2)	0.21863 (16)	−0.04271 (16)	0.0194 (9)	
C7	−0.2484 (3)	0.14164 (16)	−0.01047 (18)	0.0246 (10)	
C8	−0.3270 (3)	0.21438 (17)	−0.13518 (17)	0.0239 (10)	
C9	−0.3688 (3)	0.25190 (19)	−0.00383 (18)	0.0285 (11)	
C10	−0.1495 (3)	0.36663 (16)	−0.03135 (17)	0.0222 (10)	
C11	−0.0270 (3)	0.39435 (17)	−0.03689 (18)	0.0312 (12)	
C12	−0.2398 (3)	0.38786 (17)	−0.11023 (18)	0.0312 (12)	
C13	−0.1813 (3)	0.40295 (18)	0.04066 (18)	0.0306 (12)	
C14	0.0926 (3)	0.02904 (16)	0.19525 (18)	0.0225 (10)	
C15	−0.0099 (3)	0.02794 (19)	0.11757 (19)	0.0360 (12)	
C16	0.1749 (3)	−0.03522 (17)	0.1940 (2)	0.0352 (13)	
C17	0.0398 (3)	0.02424 (19)	0.2676 (2)	0.0385 (13)	
C18	0.2845 (3)	0.13803 (17)	0.28446 (18)	0.0270 (11)	
C19	0.3647 (4)	0.1961 (2)	0.2623 (3)	0.0712 (19)	
C20	0.3635 (3)	0.07417 (18)	0.31918 (19)	0.0335 (12)	
C21	0.2270 (4)	0.1694 (2)	0.3455 (2)	0.0595 (17)	
O1	0.4581 (2)	0.03040 (16)	0.12665 (16)	0.0561 (11)	
C22	0.5077 (3)	−0.03355 (19)	0.1672 (2)	0.0361 (13)	
C23	0.6257 (3)	−0.0110 (2)	0.21980 (19)	0.0353 (12)	
C24	0.6682 (3)	0.04216 (19)	0.1660 (2)	0.0376 (13)	
C25	0.5443 (5)	0.0679 (4)	0.1037 (5)	0.028 (2)*	0.526 (14)
C25'	0.5681 (7)	0.0877 (5)	0.1459 (7)	0.044 (3)*	0.474 (14)
O2	0.5419 (3)	0.39891 (17)	0.14239 (18)	0.0699 (13)	
C26	0.4652 (4)	0.4329 (2)	0.0740 (2)	0.0598 (18)	
C27	0.3497 (4)	0.3968 (2)	0.0567 (2)	0.0526 (17)	
C28	0.3868 (3)	0.3206 (2)	0.0795 (2)	0.0366 (13)	
C29	0.4875 (3)	0.3300 (2)	0.1544 (2)	0.0425 (14)	
H1c2	0.059049	0.188431	−0.172261	0.0235*	
H1c3	0.238693	0.123717	−0.137222	0.0277*	
H1c4	0.321964	0.088942	−0.002941	0.0325*	
H1c7	−0.186839	0.121666	−0.031736	0.0295*	
H2c7	−0.222058	0.142792	0.047401	0.0295*	
H3c7	−0.318525	0.11225	−0.026886	0.0295*	
H1c8	−0.271293	0.189676	−0.158223	0.0287*	
H2c8	−0.401014	0.188474	−0.147807	0.0287*	
H3c8	−0.340002	0.262352	−0.157041	0.0287*	
H1c9	−0.389071	0.299547	−0.025156	0.0341*	
H2c9	−0.438911	0.222295	−0.015517	0.0341*	
H3c9	−0.336027	0.254713	0.053552	0.0341*	
H1c11	−0.008889	0.374301	−0.083898	0.0375*	
H2c11	−0.028618	0.446122	−0.040693	0.0375*	
H3c11	0.032869	0.379861	0.010465	0.0375*	
H1c12	−0.219226	0.364764	−0.154818	0.0375*	
H2c12	−0.31787	0.372767	−0.10837	0.0375*	
H3c12	−0.238838	0.439353	−0.116916	0.0375*	
H1c13	−0.122777	0.390579	0.089545	0.0367*	
H2c13	−0.182949	0.454438	0.03357	0.0367*	
H3c13	−0.258094	0.386533	0.04391	0.0367*	
H1c15	0.021899	0.031633	0.07147	0.0432*	
H2c15	−0.053403	−0.016472	0.11484	0.0432*	
H3c15	−0.062181	0.06798	0.117963	0.0432*	
H1c16	0.235551	−0.037166	0.244141	0.0423*	
H2c16	0.129566	−0.079164	0.186929	0.0423*	
H3c16	0.211316	−0.029613	0.150251	0.0423*	
H1c17	0.102692	0.024961	0.316543	0.0462*	
H2c17	−0.012172	0.064643	0.266956	0.0462*	
H3c17	−0.004476	−0.019893	0.264691	0.0462*	
H1c19	0.406858	0.175825	0.226307	0.0855*	
H2c19	0.316768	0.235825	0.23631	0.0855*	
H3c19	0.420678	0.212855	0.31042	0.0855*	
H1c20	0.397018	0.054208	0.27842	0.0402*	
H2c20	0.426309	0.090032	0.364147	0.0402*	
H3c20	0.316983	0.037944	0.33691	0.0402*	
H1c21	0.180491	0.2108	0.32283	0.0714*	
H2c21	0.176437	0.133785	0.360104	0.0714*	
H3c21	0.287371	0.183707	0.392693	0.0714*	
H1c22	0.519189	−0.068374	0.128432	0.0433*	
H2c22	0.458372	−0.050564	0.200205	0.0433*	
H1c23	0.678176	−0.051946	0.230347	0.0424*	
H2c23	0.614582	0.013804	0.266395	0.0424*	
H1c24	0.705351	0.082712	0.197661	0.0451*	0.526 (14)
H2c24	0.716077	0.017253	0.136788	0.0451*	0.526 (14)
H1c24'	0.735268	0.068596	0.197649	0.0451*	0.4744
H2c24'	0.676461	0.017733	0.118449	0.0451*	0.4744
H1c25	0.544358	0.054399	0.049694	0.0339*	0.526 (14)
H2c25	0.53318	0.118777	0.110279	0.0339*	0.526 (14)
H1c25'	0.567881	0.114191	0.097713	0.0531*	0.4744
H2c25'	0.565383	0.116462	0.191882	0.0531*	0.4744
H1c26	0.497915	0.428116	0.028376	0.0717*	
H2c26	0.455711	0.482953	0.085831	0.0717*	
H1c27	0.312275	0.399219	−0.000187	0.0631*	
H2c27	0.304979	0.415078	0.092141	0.0631*	
H1c28	0.416345	0.298984	0.037768	0.044*	
H2c28	0.32261	0.295283	0.092902	0.044*	
H1c29	0.456057	0.333414	0.200692	0.051*	
H2c29	0.544145	0.291762	0.157523	0.051*	
Br1'	−0.1686 (6)	0.2563 (4)	0.2072 (4)	0.025 (2)*	0.0569 (14)
Br2'	0.1292 (11)	0.3624 (6)	0.1693 (8)	0.047 (4)*	0.0569 (14)
Mn1'	−0.0068 (11)	0.2547 (7)	0.1291 (8)	0.034 (4)*	0.0569 (14)
H1n2	−0.080 (3)	0.2446 (15)	−0.1241 (18)	0.016 (8)*	
H1n3	0.293 (3)	0.0903 (18)	0.126 (2)	0.038 (11)*	
O	0.072 (3)	0.167 (2)	0.198 (2)	0.029 (9)*	0.0569 (14)

### Atomic displacement parameters (Å^2^)

**Table:**

	*U*^11^	*U*^22^	*U*^33^	*U*^12^	*U*^13^	*U*^23^
Br1	0.01954 (18)	0.0280 (3)	0.01392 (16)	−0.00322 (16)	0.00659 (12)	−0.00286 (15)
Br2	0.0256 (2)	0.0263 (3)	0.0174 (2)	−0.0104 (2)	0.00460 (14)	−0.0030 (2)
Mn1	0.0145 (3)	0.0199 (3)	0.0096 (2)	−0.0017 (2)	0.00218 (19)	0.0000 (2)
P1	0.0152 (4)	0.0205 (4)	0.0121 (4)	0.0015 (3)	0.0039 (3)	−0.0017 (3)
P2	0.0156 (4)	0.0239 (4)	0.0147 (4)	0.0000 (3)	0.0037 (3)	0.0019 (3)
N1	0.0155 (12)	0.0254 (14)	0.0126 (12)	0.0011 (10)	0.0048 (10)	0.0028 (11)
N2	0.0168 (13)	0.0258 (14)	0.0063 (12)	0.0056 (10)	0.0036 (10)	0.0022 (11)
N3	0.0151 (14)	0.055 (2)	0.0223 (14)	0.0153 (14)	0.0074 (11)	0.0184 (14)
C1	0.0154 (14)	0.0152 (15)	0.0196 (15)	−0.0014 (12)	0.0028 (12)	0.0021 (13)
C2	0.0194 (15)	0.0230 (17)	0.0161 (15)	0.0029 (13)	0.0044 (12)	0.0025 (13)
C3	0.0228 (16)	0.0299 (18)	0.0194 (15)	0.0035 (14)	0.0108 (13)	0.0038 (15)
C4	0.0179 (16)	0.037 (2)	0.0287 (17)	0.0097 (14)	0.0107 (13)	0.0112 (16)
C5	0.0171 (15)	0.0278 (17)	0.0227 (16)	0.0029 (13)	0.0073 (12)	0.0094 (14)
C6	0.0144 (14)	0.0272 (17)	0.0156 (14)	−0.0003 (13)	0.0023 (11)	−0.0022 (14)
C7	0.0197 (16)	0.0281 (18)	0.0246 (16)	−0.0085 (13)	0.0033 (13)	−0.0034 (15)
C8	0.0196 (16)	0.0291 (18)	0.0203 (15)	0.0004 (14)	0.0001 (12)	−0.0020 (15)
C9	0.0194 (17)	0.044 (2)	0.0236 (17)	0.0002 (15)	0.0086 (14)	−0.0023 (16)
C10	0.0296 (17)	0.0209 (16)	0.0171 (15)	0.0021 (13)	0.0079 (13)	−0.0027 (14)
C11	0.043 (2)	0.0215 (18)	0.0297 (18)	−0.0028 (15)	0.0111 (16)	0.0029 (16)
C12	0.039 (2)	0.0241 (19)	0.0276 (17)	0.0087 (15)	0.0031 (15)	0.0012 (16)
C13	0.039 (2)	0.0277 (19)	0.0238 (16)	0.0080 (16)	0.0049 (15)	−0.0078 (16)
C14	0.0206 (16)	0.0198 (16)	0.0268 (16)	−0.0021 (13)	0.0057 (13)	−0.0006 (14)
C15	0.0286 (19)	0.033 (2)	0.042 (2)	−0.0101 (16)	0.0004 (16)	−0.0034 (19)
C16	0.0298 (19)	0.0210 (18)	0.054 (2)	0.0031 (15)	0.0094 (17)	−0.0070 (18)
C17	0.037 (2)	0.033 (2)	0.053 (2)	0.0011 (17)	0.0253 (18)	0.015 (2)
C18	0.0270 (17)	0.0264 (18)	0.0201 (16)	0.0010 (14)	−0.0072 (13)	0.0036 (14)
C19	0.050 (3)	0.057 (3)	0.074 (3)	−0.028 (2)	−0.043 (2)	0.033 (3)
C20	0.0285 (19)	0.036 (2)	0.0296 (18)	0.0068 (16)	−0.0047 (14)	0.0033 (17)
C21	0.058 (3)	0.067 (3)	0.036 (2)	0.032 (2)	−0.0183 (19)	−0.029 (2)
O1	0.0181 (13)	0.089 (2)	0.0629 (17)	0.0169 (14)	0.0140 (12)	0.0461 (17)
C22	0.0283 (19)	0.037 (2)	0.044 (2)	−0.0058 (16)	0.0101 (16)	−0.0047 (18)
C23	0.0240 (18)	0.051 (2)	0.0291 (18)	0.0091 (16)	0.0042 (14)	−0.0006 (18)
C24	0.0268 (18)	0.039 (2)	0.051 (2)	−0.0083 (16)	0.0163 (17)	−0.0103 (19)
O2	0.0551 (19)	0.079 (2)	0.068 (2)	−0.0295 (17)	0.0026 (16)	−0.0065 (19)
C26	0.089 (3)	0.056 (3)	0.034 (2)	−0.020 (3)	0.015 (2)	0.004 (2)
C27	0.069 (3)	0.051 (3)	0.038 (2)	−0.006 (2)	0.015 (2)	−0.009 (2)
C28	0.035 (2)	0.042 (2)	0.0339 (19)	−0.0001 (17)	0.0119 (16)	−0.0092 (18)
C29	0.047 (2)	0.053 (3)	0.0293 (19)	−0.003 (2)	0.0123 (17)	0.0025 (19)

### Geometric parameters (Å, º)

**Table:**

Br1—Mn1	2.5677 (7)	C15—H1c15	0.96
Br1—N2^i^	3.625 (2)	C15—H2c15	0.96
Br1—Br1'	0.845 (8)	C15—H3c15	0.96
Br1—Mn1'	2.398 (15)	C16—H1c16	0.96
Br2—Mn1	2.4968 (9)	C16—H2c16	0.96
Br2—Br2'	0.446 (12)	C16—H3c16	0.96
Br2—Mn1'	2.258 (12)	C17—H1c17	0.96
Mn1—P1	2.6443 (9)	C17—H2c17	0.96
Mn1—P2	2.6395 (10)	C17—H3c17	0.96
Mn1—N1	2.355 (3)	C18—C19	1.535 (6)
Mn1—Br1'	2.918 (7)	C18—C20	1.520 (4)
Mn1—Br2'	2.820 (12)	C18—C21	1.501 (5)
Mn1—Mn1'	0.597 (13)	C19—H1c19	0.96
Mn1—O	1.65 (4)	C19—H2c19	0.96
P1—N2	1.685 (3)	C19—H3c19	0.96
P1—C6	1.870 (3)	C20—H1c20	0.96
P1—C10	1.867 (3)	C20—H2c20	0.96
P1—Mn1'	2.453 (12)	C20—H3c20	0.96
P2—N3	1.690 (3)	C21—H1c21	0.96
P2—C14	1.872 (3)	C21—H2c21	0.96
P2—C18	1.869 (3)	C21—H3c21	0.96
P2—O	1.38 (4)	O1—C22	1.417 (4)
N1—C1	1.357 (3)	O1—C25	1.361 (8)
N1—C5	1.355 (4)	O1—C25'	1.629 (9)
N2—C1	1.379 (4)	C22—C23	1.493 (4)
N2—Br1'^ii^	3.629 (6)	C22—H1c22	0.96
N2—H1n2	0.83 (3)	C22—H2c22	0.96
N3—C5	1.382 (4)	C23—C24	1.518 (5)
N3—H1n3	0.78 (3)	C23—H1c23	0.96
C1—C2	1.391 (4)	C23—H2c23	0.96
C2—C3	1.378 (4)	C24—C25	1.628 (7)
C2—H1c2	0.96	C24—C25'	1.407 (9)
C3—C4	1.375 (4)	C24—H1c24	0.96
C3—H1c3	0.96	C24—H2c24	0.96
C4—C5	1.394 (5)	C24—H1c24'	0.96
C4—H1c4	0.96	C24—H2c24'	0.96
C6—C7	1.533 (4)	C25—C25'	0.793 (13)
C6—C8	1.543 (4)	C25—H1c25	0.96
C6—C9	1.531 (5)	C25—H2c25	0.96
C7—H1c7	0.96	C25—H1c25'	0.9125
C7—H2c7	0.96	C25'—H2c25	0.8596
C7—H3c7	0.96	C25'—H1c25'	0.96
C8—H1c8	0.96	C25'—H2c25'	0.96
C8—H2c8	0.96	O2—C26	1.423 (5)
C8—H3c8	0.96	O2—C29	1.462 (5)
C9—H1c9	0.96	C26—C27	1.462 (6)
C9—H2c9	0.96	C26—H1c26	0.96
C9—H3c9	0.96	C26—H2c26	0.96
C10—C11	1.543 (5)	C27—C28	1.496 (5)
C10—C12	1.532 (4)	C27—H1c27	0.96
C10—C13	1.535 (5)	C27—H2c27	0.96
C11—H1c11	0.96	C28—C29	1.506 (4)
C11—H2c11	0.96	C28—H1c28	0.96
C11—H3c11	0.96	C28—H2c28	0.96
C12—H1c12	0.96	C29—H1c29	0.96
C12—H2c12	0.96	C29—H2c29	0.96
C12—H3c12	0.96	H1c24—H1c24'	0.4356
C13—H1c13	0.96	H2c24—H2c24'	0.4859
C13—H2c13	0.96	H2c25—H1c25'	0.5126
C13—H3c13	0.96	Br1'—Mn1'	2.584 (17)
C14—C15	1.540 (4)	Br2'—Mn1'	2.526 (17)
C14—C16	1.531 (4)	Mn1'—O	2.08 (4)
C14—C17	1.525 (5)		
			
Mn1—Br1—N2^i^	122.86 (4)	C14—C17—H2c17	109.47
Mn1—Br1—Br1'	106.2 (5)	C14—C17—H3c17	109.47
Mn1—Br1—Mn1'	13.3 (3)	H1c17—C17—H2c17	109.47
N2^i^—Br1—Br1'	83.6 (4)	H1c17—C17—H3c17	109.47
N2^i^—Br1—Mn1'	120.9 (3)	H2c17—C17—H3c17	109.47
Br1'—Br1—Mn1'	93.0 (6)	P2—C18—C19	107.1 (2)
Mn1—Br2—Br2'	133.1 (15)	P2—C18—C20	116.4 (2)
Mn1—Br2—Mn1'	13.2 (3)	P2—C18—C21	106.3 (2)
Br2'—Br2—Mn1'	122.5 (15)	C19—C18—C20	107.2 (3)
Br1—Mn1—Br2	104.44 (3)	C19—C18—C21	108.3 (3)
Br1—Mn1—P1	97.75 (3)	C20—C18—C21	111.2 (3)
Br1—Mn1—P2	98.74 (3)	C18—C19—H1c19	109.47
Br1—Mn1—N1	146.86 (6)	C18—C19—H2c19	109.47
Br1—Mn1—Br1'	16.14 (15)	C18—C19—H3c19	109.47
Br1—Mn1—Br2'	104.6 (3)	H1c19—C19—H2c19	109.47
Br1—Mn1—Mn1'	67.0 (14)	H1c19—C19—H3c19	109.47
Br1—Mn1—O	73.0 (15)	H2c19—C19—H3c19	109.47
Br2—Mn1—P1	103.82 (3)	C18—C20—H1c20	109.47
Br2—Mn1—P2	104.95 (3)	C18—C20—H2c20	109.47
Br2—Mn1—N1	108.70 (6)	C18—C20—H3c20	109.47
Br2—Mn1—Br1'	94.86 (14)	H1c20—C20—H2c20	109.47
Br2—Mn1—Br2'	6.6 (2)	H1c20—C20—H3c20	109.47
Br2—Mn1—Mn1'	60.0 (12)	H2c20—C20—H3c20	109.47
Br2—Mn1—O	104.3 (12)	C18—C21—H1c21	109.47
P1—Mn1—P2	141.86 (3)	C18—C21—H2c21	109.47
P1—Mn1—N1	74.02 (5)	C18—C21—H3c21	109.47
P1—Mn1—Br1'	87.62 (11)	H1c21—C21—H2c21	109.47
P1—Mn1—Br2'	97.3 (2)	H1c21—C21—H3c21	109.47
P1—Mn1—Mn1'	65.1 (12)	H2c21—C21—H3c21	109.47
P1—Mn1—O	151.8 (12)	C22—O1—C25	109.4 (3)
P2—Mn1—N1	73.40 (6)	C22—O1—C25'	104.2 (4)
P2—Mn1—Br1'	114.11 (14)	C25—O1—C25'	29.0 (5)
P2—Mn1—Br2'	111.3 (2)	O1—C22—C23	104.9 (3)
P2—Mn1—Mn1'	152.8 (12)	O1—C22—H1c22	109.47
P2—Mn1—O	26.7 (15)	O1—C22—H2c22	109.47
N1—Mn1—Br1'	152.83 (13)	C23—C22—H1c22	109.47
N1—Mn1—Br2'	108.3 (3)	C23—C22—H2c22	109.47
N1—Mn1—Mn1'	131.2 (13)	H1c22—C22—H2c22	113.73
N1—Mn1—O	98.9 (14)	C22—C23—C24	102.0 (3)
Br1'—Mn1—Br2'	93.6 (3)	C22—C23—H1c23	109.47
Br1'—Mn1—Mn1'	51.0 (14)	C22—C23—H2c23	109.47
Br1'—Mn1—O	87.8 (15)	C24—C23—H1c23	109.47
Br2'—Mn1—Mn1'	55.0 (12)	C24—C23—H2c23	109.47
Br2'—Mn1—O	110.8 (12)	H1c23—C23—H2c23	116.01
Mn1'—Mn1—O	129.6 (19)	C23—C24—C25	102.4 (4)
Mn1—P1—N2	98.90 (8)	C23—C24—C25'	99.0 (5)
Mn1—P1—C6	118.03 (9)	C23—C24—H1c24	109.47
Mn1—P1—C10	118.04 (9)	C23—C24—H2c24	109.47
Mn1—P1—Mn1'	12.8 (3)	C23—C24—H1c24'	109.47
N2—P1—C6	102.01 (13)	C23—C24—H2c24'	109.47
N2—P1—C10	104.40 (14)	C25—C24—C25'	29.2 (5)
N2—P1—Mn1'	109.4 (3)	C25—C24—H1c24	109.47
C6—P1—C10	111.87 (13)	C25—C24—H2c24	109.47
C6—P1—Mn1'	120.6 (3)	C25—C24—H1c24'	132.27
C10—P1—Mn1'	107.3 (3)	C25—C24—H2c24'	81.22
Mn1—P2—N3	97.73 (10)	C25'—C24—H1c24	83.85
Mn1—P2—C14	117.28 (9)	C25'—C24—H2c24	135.73
Mn1—P2—C18	118.77 (10)	C25'—C24—H1c24'	109.47
Mn1—P2—O	32.5 (16)	C25'—C24—H2c24'	109.47
N3—P2—C14	104.08 (15)	H1c24—C24—H2c24	115.69
N3—P2—C18	103.39 (14)	H1c24—C24—H1c24'	26.22
N3—P2—O	130.2 (16)	H1c24—C24—H2c24'	135.91
C14—P2—C18	111.99 (13)	H2c24—C24—H1c24'	92.42
C14—P2—O	102.6 (16)	H2c24—C24—H2c24'	29.32
C18—P2—O	104.4 (14)	H1c24'—C24—H2c24'	118.23
Mn1—N1—C1	121.76 (19)	O1—C25—C24	104.7 (5)
Mn1—N1—C5	120.72 (18)	O1—C25—C25'	94.5 (11)
C1—N1—C5	117.0 (3)	O1—C25—H1c25	109.47
Br1^ii^—N2—P1	124.14 (10)	O1—C25—H2c25	109.47
Br1^ii^—N2—C1	109.31 (18)	O1—C25—H1c25'	141.04
Br1^ii^—N2—Br1'^ii^	13.37 (12)	C24—C25—C25'	59.8 (7)
Br1^ii^—N2—H1n2	6 (2)	C24—C25—H1c25	109.47
P1—N2—C1	126.3 (2)	C24—C25—H2c25	109.47
P1—N2—Br1'^ii^	124.64 (15)	C24—C25—H1c25'	96.15
P1—N2—H1n2	123 (2)	C25'—C25—H1c25	155.83
C1—N2—Br1'^ii^	108.5 (2)	C25'—C25—H2c25	57.78
C1—N2—H1n2	111 (2)	C25'—C25—H1c25'	68.08
Br1'^ii^—N2—H1n2	8.5 (19)	H1c25—C25—H2c25	113.82
P2—N3—C5	124.8 (2)	H1c25—C25—H1c25'	93.45
P2—N3—H1n3	121 (3)	H2c25—C25—H1c25'	31.65
C5—N3—H1n3	114 (3)	O1—C25'—C24	102.6 (6)
N1—C1—N2	118.0 (3)	O1—C25'—C25	56.4 (8)
N1—C1—C2	123.2 (3)	O1—C25'—H2c25	95.19
N2—C1—C2	118.7 (2)	O1—C25'—H1c25'	109.47
C1—C2—C3	117.8 (3)	O1—C25'—H2c25'	109.47
C1—C2—H1c2	121.12	C24—C25'—C25	91.1 (9)
C3—C2—H1c2	121.12	C24—C25'—H2c25	141.59
C2—C3—C4	120.9 (3)	C24—C25'—H1c25'	109.47
C2—C3—H1c3	119.57	C24—C25'—H2c25'	109.47
C4—C3—H1c3	119.57	C25—C25'—H2c25	70.88
C3—C4—C5	117.9 (3)	C25—C25'—H1c25'	61.87
C3—C4—H1c4	121.04	C25—C25'—H2c25'	157.99
C5—C4—H1c4	121.05	H2c25—C25'—H1c25'	32.13
N1—C5—N3	117.8 (3)	H2c25—C25'—H2c25'	95.86
N1—C5—C4	123.0 (3)	H1c25'—C25'—H2c25'	115.59
N3—C5—C4	119.1 (3)	C26—O2—C29	107.4 (3)
P1—C6—C7	104.40 (17)	O2—C26—C27	108.1 (3)
P1—C6—C8	114.1 (2)	O2—C26—H1c26	109.47
P1—C6—C9	110.4 (2)	O2—C26—H2c26	109.47
C7—C6—C8	108.2 (2)	C27—C26—H1c26	109.47
C7—C6—C9	109.0 (3)	C27—C26—H2c26	109.47
C8—C6—C9	110.4 (2)	H1c26—C26—H2c26	110.78
C6—C7—H1c7	109.47	C26—C27—C28	101.0 (3)
C6—C7—H2c7	109.47	C26—C27—H1c27	109.47
C6—C7—H3c7	109.47	C26—C27—H2c27	109.47
H1c7—C7—H2c7	109.47	C28—C27—H1c27	109.47
H1c7—C7—H3c7	109.47	C28—C27—H2c27	109.47
H2c7—C7—H3c7	109.47	H1c27—C27—H2c27	116.82
C6—C8—H1c8	109.47	C27—C28—C29	102.8 (3)
C6—C8—H2c8	109.47	C27—C28—H1c28	109.47
C6—C8—H3c8	109.47	C27—C28—H2c28	109.47
H1c8—C8—H2c8	109.47	C29—C28—H1c28	109.47
H1c8—C8—H3c8	109.47	C29—C28—H2c28	109.47
H2c8—C8—H3c8	109.47	H1c28—C28—H2c28	115.39
C6—C9—H1c9	109.47	O2—C29—C28	104.5 (3)
C6—C9—H2c9	109.47	O2—C29—H1c29	109.47
C6—C9—H3c9	109.47	O2—C29—H2c29	109.47
H1c9—C9—H2c9	109.47	C28—C29—H1c29	109.47
H1c9—C9—H3c9	109.47	C28—C29—H2c29	109.47
H2c9—C9—H3c9	109.47	H1c29—C29—H2c29	114.04
P1—C10—C11	106.5 (2)	C24—H1c24—H1c24'	76.89
P1—C10—C12	116.3 (2)	C24—H2c24—H2c24'	75.34
P1—C10—C13	107.6 (2)	C24—H1c24'—H1c24	76.89
C11—C10—C12	107.0 (3)	C24—H2c24'—H2c24	75.34
C11—C10—C13	109.1 (2)	C25—H2c25—C25'	51.33
C12—C10—C13	110.2 (2)	C25—H2c25—H1c25'	69.07
C10—C11—H1c11	109.47	C25'—H2c25—H1c25'	84.79
C10—C11—H2c11	109.47	C25—H1c25'—C25'	50.05
C10—C11—H3c11	109.47	C25—H1c25'—H2c25	79.29
H1c11—C11—H2c11	109.47	C25'—H1c25'—H2c25	63.09
H1c11—C11—H3c11	109.47	Br1—Br1'—Mn1	57.7 (4)
H2c11—C11—H3c11	109.47	Br1—Br1'—N2^i^	83.0 (4)
C10—C12—H1c12	109.47	Br1—Br1'—Mn1'	67.9 (5)
C10—C12—H2c12	109.47	Mn1—Br1'—N2^i^	112.62 (18)
C10—C12—H3c12	109.47	Mn1—Br1'—Mn1'	10.3 (3)
H1c12—C12—H2c12	109.47	N2^i^—Br1'—Mn1'	115.2 (3)
H1c12—C12—H3c12	109.47	Br2—Br2'—Mn1	40.3 (13)
H2c12—C12—H3c12	109.47	Br2—Br2'—Mn1'	48.9 (14)
C10—C13—H1c13	109.47	Mn1—Br2'—Mn1'	11.2 (3)
C10—C13—H2c13	109.47	Br1—Mn1'—Br2	118.6 (6)
C10—C13—H3c13	109.47	Br1—Mn1'—Mn1	99.8 (15)
H1c13—C13—H2c13	109.47	Br1—Mn1'—P1	108.1 (5)
H1c13—C13—H3c13	109.47	Br1—Mn1'—Br1'	19.1 (2)
H2c13—C13—H3c13	109.47	Br1—Mn1'—Br2'	120.1 (6)
P2—C14—C15	103.2 (2)	Br1—Mn1'—O	71.0 (12)
P2—C14—C16	114.9 (2)	Br2—Mn1'—Mn1	106.8 (13)
P2—C14—C17	110.2 (2)	Br2—Mn1'—P1	118.4 (6)
C15—C14—C16	108.9 (2)	Br2—Mn1'—Br1'	111.1 (6)
C15—C14—C17	108.5 (3)	Br2—Mn1'—Br2'	8.6 (3)
C16—C14—C17	110.7 (3)	Br2—Mn1'—O	99.5 (11)
C14—C15—H1c15	109.47	Mn1—Mn1'—P1	102.2 (13)
C14—C15—H2c15	109.47	Mn1—Mn1'—Br1'	118.7 (15)
C14—C15—H3c15	109.47	Mn1—Mn1'—Br2'	113.8 (14)
H1c15—C15—H2c15	109.47	Mn1—Mn1'—O	37.6 (15)
H1c15—C15—H3c15	109.47	P1—Mn1'—Br1'	99.9 (5)
H2c15—C15—H3c15	109.47	P1—Mn1'—Br2'	111.0 (6)
C14—C16—H1c16	109.47	P1—Mn1'—O	133.6 (11)
C14—C16—H2c16	109.47	Br1'—Mn1'—Br2'	109.9 (6)
C14—C16—H3c16	109.47	Br1'—Mn1'—O	89.2 (13)
H1c16—C16—H2c16	109.47	Br2'—Mn1'—O	108.1 (11)
H1c16—C16—H3c16	109.47	Mn1—O—P2	121 (3)
H2c16—C16—H3c16	109.47	Mn1—O—Mn1'	12.7 (5)
C14—C17—H1c17	109.47	P2—O—Mn1'	132 (3)

Symmetry codes: (i) *x*, −*y*+1/2, *z*+1/2; (ii) *x*, −*y*+1/2, *z*−1/2.

### Hydrogen-bond geometry (Å, º)

**Table:**

*D*—H···*A*	*D*—H	H···*A*	*D*···*A*	*D*—H···*A*
N2—H1N2···Br1^ii^	0.83 (3)	2.80 (3)	3.625 (2)	173 (2)
N2—H1N2···Br1′^ii^	0.83 (3)	2.81 (3)	3.629 (7)	169 (3)
N3—H1N3···O1	0.78 (4)	2.22 (4)	2.990 (4)	171 (3)

Symmetry code: (ii) *x*, −*y*+1/2, *z*−1/2.
